# FACTORS ASSOCIATED WITH COMMUNITY-DWELLING OLDER ADULTS’ VIEWS OF DEPRESCRIBING: A PILOT STUDY

**DOI:** 10.1093/geroni/igae098.3012

**Published:** 2024-12-31

**Authors:** Hyunjin Noh, Lewis Lee, Haelim Jeong, Lauryn Emrick, Madison Hasnani, Wendy Waddell

**Affiliations:** University of Alabama, Tuscaloosa, Alabama, United States; University of Alabama, Tuscaloosa, Alabama, United States; University of Alabama, Tuscaloosa, Alabama, United States; University of Alabama, Tuscaloosa, Alabama, United States; University of Alabama, Tuscaloosa, Alabama, United States; University of Alabama, Tuscaloosa, Alabama, United States

## Abstract

Ample evidence exists on the risks of adverse drug interactions, calling for reducing or stopping ineffective or harmful medications (i.e., describing). However, limited research exists on the views of deprescribing and associated factors among older adults. To fill this gap, this study aimed to examine factors associated with the views of deprescribing among older adults living with multimorbidity and chronic pain. Using purposeful sampling, 132 participants were recruited for a phone survey, who were 65+, living outside of nursing homes, cognitively intact, have two or more chronic illnesses and chronic pain, and take medications for the illnesses, including pain medicine. The revised patient attitudes towards deprescribing (rPATD) questionnaire encompassed five dependent variables: Burden, appropriateness, concerns about stopping, involvement, and willingness to discontinue regular medications. Independent variables included sociodemographic/pain-related variables. A series of regression analyses showed a positive association between the number of medications and the burden factor. Pain scores were positively associated with the belief of medication-induced side effects. The number of chronic health conditions was inversely associated with concerns about cessation, while pain scores were positively related to the same outcome. Age and race were negatively related to the involvement factor. Lastly, gender and marital status were significantly related to the inclination to discontinue regular medications. Findings suggest targeted education efforts for those with a higher number of illnesses, pain, and medications to demystify their concerns about deprescribing and reduce their medication-related burden. Furthermore, advocacy efforts are needed to support older and Black older adults’ involvement in medication decision-making.

